# Therapœia

**Published:** 1825-10-01

**Authors:** 


### Therapœia.

---

*Pharmaca nulla valent, nisi quæ sint commoda causis.*

---

1. *Chorea.** We have always considered it much more curious than useful, to record the infinity of eccentric motions and preposterous attitudes exhibited by patients labouring under the higher grades of chorea. We remember (and so must many of our readers) a most circumstantial detail of this kind, published by Dr. Watt, in one of the early volumes of the Medico-chirurgical Transactions, under the title of "Periodical Jactitation." Among many other freaks of this lively young girl, one was that of spinning round like a top—and another, that of *standing* (Hibernice) *on her head.*

---

* Mr. Robert Hunter—Dr. Thomas Jeffreys. Ed. Journal, April, 1825.

Mr. Hunter's patient was also a young female, whose choreal propensity consisted in rotating the head, with a velocity that would have left Harlequin far behind. "The rotatory motions of the head now became furious and alarming. They were executed with such immense rapidity, that it was difficult even for the eye to follow them." The young woman did not appear to derive much pleasure from these rotatory fits—on the contray, she averred that she would submit to any operation, however painful—that she would even "allow herself to be opened alive," to get rid of so distressing a malady. She had been some years ill, and the chorea appeared to have been preceded by some uterine and hepatic derangement. She had been in the Infirmary of Glasgow, and under various medical practitioners, without deriving any advantage from medicine.

Mr. Hunter ordered the head to be shaved, and tartar emetic ointment to be rubbed on the scalp, and also along the spine. As soon as a large crop of pustules came out, the rotation was greatly diminished, and, by steady perseverance in the application, together with tonics, she entirely recovered. This plan of treatment, as far as regards the antimoniated ointment, is novel, and deserves attention.

In the same Journal, and in immediate succession, we have two cases, related by Dr. Jeffreys of Liverpool. These cases are (ut mos est) preceded by historical notices, from Paracelsus to Hamilton—together with a kind of regular dissertation on the disease in general, and also on the nature of spasm. These we must pass over. The first case related is that of a young lady, where the remedies were so multitudinous that it is difficult to say which had the predominant merit in curing the disease—or whether medicine had any other share in the cure than that of nauseating the stomach, and preventing too much ingesta. The principal remedies employed were, antispasmodics, purgatives, and tonics. She recovered, though slowly.

The second case is a melancholy one. The patient was a professional man (an attorney) aged 54, when first he was attacked with the complaint. He had hitherto been healthy and vigorous. He first became affected with tremors of the hands, which increased to a troublesome degree, but unaccompanied with pain, except the weariness occasioned by constant agitation. The Provincial physicians having all failed to give any relief, recourse was had to those of the Metropolis, "where (as Samuel Johnson said) flows the great tide of human existence," and where it is often vainly hoped that the tide of existence can be altered in its course! Application was made to Dr. Meyers, (query, the Jew or the German?) who suspected the disease might arise from atonic gout. Temperance, exercise, tonics, and antispasmodics were recommended, and failed. Dr. Gregory (the great) was next consulted, and added opium, hemlock and gamboge, to the plan of Dr. Meyers. These failed also. Mercury was then tried, but no salivation could be induced by any possible means. This was a strong indication of disease of the brain, which is well-known to prevent salivation. He dragged on a wretched existence for 25 *years* in this state, and died at the age of 70, not of the chorea, if so it may be called, but of a slight febrile attack.

---

2. *Poisoning by Arsenic and Sublimate.* A young pharmaceutical student, of sanguineous temperament and robust constitution, mixed up, in a fit of despair, a drachm of arsenic and the same quantity of oxymuriate of mercury, into ten boluses, nine of which he swallowed on the spot, and retained the tenth, to shew the kind of poison he had taken, convinced that his death would be very sudden from the dose taken. It was not so, however, and in the space of five minutes, on feeling a burning heat in his bowels, he repented of the rash act, and quickly swallowed eighteen grains of ipecacuan, which caused the rejection of a part of the poison. The fear of death now induced him to cry out furiously for aid, and M. Julia-Fontenelle was instantly on the spot. He found the patient rolling on the floor, the picture of misery and terror. He frankly avowed all that had passed. Abundance of warm water was taken into the stomach, and black matters were vomited forth. These, on subsequent analysis, presented the poisonous substances swallowed. Several other medical gentlemen were now summoned to the assistance of M. Fontenelle. Warm milk was thrown into the stomach in large quantities, and also into the bowels by injection. The patient experienced excruciating pains, and insatiable thirst. In four hours from the accident he had convulsions, and appeared at the point of death. In two hours more, however, he appeared a little better. A diarrhœa came on, which lasted eight days. Sugar and milk were assiduously administered, together with whites of eggs. *Next day*, face flushed, and countenance wild—abdomen extremely painful—constant vomiting, diarrhœa, and insatiable thirst, with convulsive twitchings. Milk still for drink—cataplasms to the abdomen—24 leeches to the chest. *Third day*, the symptoms ameliorated—same treatment. *Fourth day*, the convulsive movements more strong—facies Hippocratica—frequent vomiting of green matters—violent cholic and delirium—bled largely from the arm—warm bath—stimulating plaster to the epigastrium—large draughts of whey. *Fifth day*, no better—large blister to the breast—sinapisms to the legs. *Sixth day*, the vomitings less frequent—delirium milder. During the next two days, the symptoms still more favourable. Mercurial ptyalism and ulcers in the mouth—extreme debility. By the 12th day, he began to convalesce, and by the end of three weeks he was able to undertake a journey of 150 miles to his native air. He has since enjoyed good health, and is become robust. *Archives Generales.*

---

3. *Treatment of Scrofulous Ulcerations.** Mr. Rennie wishes to draw the attention of the faculty to certain topical applications in scrofulous ulcerations. Having witnessed the beneficial effects of *pitch*, as

* A. Rennie, Esq. Med. Repos. March, 1825.

a stimulating and detergent application to foul and indolent sores of this nature, he was led to form it into a plaster of such consistency as to combine pressure with the peculiar qualities of the pitch.

"The following compositions, after experimental trials made at my suggestion,* have been found to combine, in the greatest perfection, the requisite qualities as above described:—

I. *Medium.*

"℞ Picis Nigræ, p. j.
—— Liquidæ, p. iss.
—— Resinæ, p. ij. M. ft. emplastrum.

II. *Soft.*

"℞ Picis Liquid. p. iss.
—— Nigr. p. j.
—— Resinæ, p. j. M. ft. emp.

III. *Hard.*

"℞ Picis Liq. p. iij.
—— Resinæ, p. iv. M.

IV. *Solid.*

"℞ Picis Nigræ, p. ij.

Emp. Resinæ, p. j.

—— Ceræ, p. iij. M.

"To be heated and spread at the time of application, not, however, too thinly. The best thickness seems to be from one to two lines." 194.

With regard to constitutional treatment in cases of scrofula, Mr. R. wishes to remark that, in his experience, the exhibition of calomel, in minute doses, frequently repeated, is an ineffectual and unsuitable method of procedure. "The mercurial not being in doses sufficiently active to carry itself off, or clear out offending matters, remains in the system exciting the mercurial irritation; and, notwithstanding a protracted course, accumulations of morbid fæcal matter are frequently found to exist." We are not advocates for allowing fæcal accumulations to take place; but we have seen so much benefit from small doses of mercury in improving the secretions, and in that way improving the general health, that we cannot consent to abandon the procedure, notwithstanding the respect we entertain for Mr. Rennie's opinions. We generally give some smart but unirritating purgative, every third or fourth day, while pursuing the alterative course, and thus avoid the chance of fæcal accumulations. But we must hear Mr. Rennie.

"It appears to me that the greatest benefit is derived from calomel, when prescribed in adequate doses to excite considerable activity both of the secreting and peristaltic functions, and followed, within eight or twelve hours, by such other aperients as completely to evacuate the intestinal canal.

* By Messrs. Waugh, Chemists, Regent Street.

"This active operation will not bear to be repeated oftener than once in four to six days. One advantage is gained by delay, that the constitution has time completely to recover from the mercurial irritation and the depressing effect of copious evacuation, whilst morbid accumulations are sufficiently obviated.

"So long as the discharges continue unhealthy, perseverance in aperient measures is the chief indication. To obviate unpleasant effects from the continued use of active mercurials, it is advisable, after the few first repetitions, to interpose mild doses of rhubarb and soda, or magnesia, prescribing mercurials only once in six to twelve days, as occasion requires." 196.

Our author throws out some judicious hints respecting the exhibition of mercurials in cold, and especially damp weather, in which seasons much inconvenience is often experienced from the use of calomel. It then frequently produces tormina and tenesmus, "in a manner so capricious as no foresight could anticipate." The distention of the alvine viscera, in scrofulous constitutions, is, in Mr. R.'s opinion, dependent on a peculiar disordered condition of the hepatic functions, "consisting not of acute inflammatory action, nor of permanent alteration of structure, but of vascular congestion and inactivity of the vital functions, dependent on diminished vital power, together with suspended, irregular, or depraved biliary secretion. It is in the power of correcting this condition of the alvine secretions that the beneficial effects of mercurials depend. Speaking of tonics, Mr. Rennie observes:—

"Notwithstanding the general debility so invariably attendant on the scrofulous state of the constitution, I am convinced that the administration of this class of remedies is much seldomer advantageous than is perhaps supposed. On the restoration of healthy hepatic and alvine functions, it is surprising how speedily the youthful constitution regains vigour, and every symptom of debility and relaxation disappears. As conducive to this effect, the sedative and invigorating influences of pure air, moderate exercise, equable and warm clothing, and generous diet, are unquestionably of the first importance. Where these requisites are possessed, tonic remedies are less necessary; and where these advantages are not enjoyed, they frequently do more harm than good.

"The system generally found most suitable has been to administer an alterative mercurial every sixth night, and a full purgative next morning; for two days thereafter tonics; the third evening or morning a mild aperient of rhubarb, with alkali or magnesia; then tonics for two days, or, at most, three: after which, the alterative and purgative as before." 198.

Our author has found better effects from the sulphate of quinine, in allaying irritability, and "counteracting that febrile diathesis depending on stomachic atony," than from any other tonic at present known. We cannot follow Mr. Rennie through his detail of the local treatment of scrofulous sores, but refer to the paper itself for fuller information on that point. The communication is, upon the whole, creditable to the author.

4. *Mercurial Fumigation.*[*] Nothing is more harrassing to the military surgeon in India than the treatment of chronic affections of an anomalous character. After trying various remedies without decided benefit men are discharged as incurable—and after a time, to the surprise and disgrace of the surgeon, they are often seen re-entering the service, free from disease, and full of energy and activity. This was particularly exemplified in our author's practice by the return of a Sepoy, after a short leave of absence, supple and active, who had left the hospital crippled and nearly helpless. "Academic honours blushed, for a moment, to be outdone by some illiterate Paracelsus of a Mahratta hamlet." It was evident in this case, that mercury had been used, in some form or other. But the Sepoy was either too ignorant, or his doctor too cunning; for Dr. G. could not ascertain that any mercury had been actually exhibited internally. He had been exposed to fire and smoke, and drank some vegetable decoction, during which his mouth had become very sore, and he himself rapidly convalescent. This and other considerations induced our author to try the effects of mercurial fumigations, for many chronic complaints, but chiefly for that *opprobrium medicorum*— chronic rheumatism. The mode in which Dr. Gibson performed this process was by putting some oxide of mercury—or, what he prefers, half a drachm of the pilula hydrargyri, on a piece of tile placed over some live embers in an earthen dish, on the ground, between the patient's legs, the patient himself being enveloped in a blanket. The mercury is thus volatilised, and the process is repeated night and morning, as long as may be deemed necessary. In venereal affections of every stage, this kind of fumigation (the ingredients being somewhat different) is in universal use among the natives, who exhibit no deforming traces either of its baleful consequences or inertness. A letter from Dr. Milne to our author corroborates the efficacy of this mode of treatment in chronic rheumatism, and the sequelæ of syphilitic and mercurial affections.

We hope our Indian brethern will profit by this communication from an intelligent and meritorious medical officer.

---

5. *Yellow Fever.*[†] Dr. Belcher sojourned for a time in the West Indies, as an assistant surgeon in His Majesty's ship Gloucester. The few observations which he has thrown together, in our respected cotemporary, on the subject of yellow fever, exhibit nothing novel—except the contradiction of some opinions that are new, and the confirmation of others that are now old. The disease, as he saw it, was not contagious —though he denies not the probability of its becoming so, under different circumstances; as "neglect of cleanliness, ill ventilation, impure atmosphere, a crowded state of the sick, &c."

---

* Dr. Gibson, Staff-Surgeon, East Indies. Med. and Phys Journal, July, 1825.

† Dr. Belcher. Ed. Journal, April, 1825.

"On looking over," says he, "the different authors who have so ably treated of this scourge, I find in none so able, so concise, and so truly faithful a description, as that given by Dr. M'Arthur, (Johnson's influence of tropical climates) the late physician to the local naval Hospital. It is a relation of facts taken from nature—from the bed side of the patient—and facts will contradict theory to the end of time."

The treatment which our author recommends is strictly depletory in the early stage of re-action—namely, by bleeding to 40 or even 80 ounces in the horizontal position, so that a decided impression might be made on the disease, without the interruption of syncope. The loss of blood was followed by the exhibition of an active purgative bolus, as twelve grains of calomel and a scruple of jalap, assisted by salts and senna. In very many cases nothing else was necessary than to keep up the catharsis. But there were other and more formidable cases, where depletion alone was not sufficient to arrest the progress of the disease.

"In all cases which were not relieved by the primary depletions, we found the most decidedly beneficial effects from the exhibition of mercury, so as to affect the mouth."

Three grains of calomel were exhibited every two or three hours till salivation was raised. This required from 24 to 36 or 40 hours. When the calomel produced irritation in the intestinal canal, it was guarded by small doses of opium.

"We found, in almost all favourable cases, as the mercurial action became established, the febrile diminished. There is something peculiar in the action which mercury exerts on the system, which renders its existence with febrile action incompatible. I cannot too strongly recommend the mercurial practice in tropical fevers, conducted in the above manner. I have seen it most beneficial, and I am confident that it has saved many a life, when proper evacuating measures had been premised. I am by no means an advocate for the wanton use or the abuse of mercury, nor have I that *horror hydrargyri*, that I am sorry to see so many of my professional brethren possess at the present day. I am not so prejudiced against its use, as to allow a fine child to expire, in *cynanche trachealis* from suffocation;—or to suffer a female of 16 or 18 to lose an eye from the effects of iritis; nor am I so prejudiced in favour of its abuse, as to *accelerate the destruction of the nasal spongy bones*, or of a penis labouring under gangrenous inflammation, accompanied with ulcerations." 255.

We believe that more spongy bones have been lost by the "*horror hydrargyri*," than by even the rash use of this mineral. The loss of reputation and the loss of practice are now beginning to check this *horror hydrargyri* which, at one time, threatened to become epidemic.

Upon the whole, Dr. Belcher's paper is creditable to his discrimination as an observant practitioner. Our readers will see that its merits consist more in the defence of old, than in the promulgation of new doctrines and practices. But even this merit is no mean or useless one.

6. *Remedies for Itch.** For the benefit of all those whom it may concern, on either side of the Tweed—or even in our good town of Berwick, we publish the following important information respecting the best, quickest, and *cheapest* modes of curing the itch. Dr. Maury has made a series of experiments to this effect, in the St. Louis Hospital—and twenty-one formulæ are given, with their results. We subjoin four of the most efficacious.

"1. "Camphorated liniment of M. Vaidy; composed of two ounces of olive or almond oil, and two drachms of camphor. Mean duration of the treatment, $13\frac{3}{10}$ days. This medicine is too expensive for habitual use at an hospital; it stains the linen; the smell is not unpleasant; it effects the cure without irritating the skin, and the itching is much relieved by the first application. It is recommended as a good remedy for private practice. The compound liniment of M. Fournier differs from the preceding only in the addition of two drachms of liquid ammonia, and the combination is favourably spoken of. The medium length of time required for the cure being reduced to $11\frac{4}{10}$ days.

"2. Sulphur pomatum of M. Helmerick: sublimed sulphur, two parts; purified potass, one part; lard, eight parts. Two frictions are made in the day, using two ounces of the pomatum for each. Mean duration of the treatment, $11\frac{7}{10}$ days. The price of this is moderate; it soils the linen from the excess of fat over the alkali; has some smell, but does not incommode the skin, and effects a speedy cure. It differs little from the "pommade sulphuro-alcaline," employed at the hospital of St. Louis.

"3. Pomatum proposed by M. Melier: subcarbonate of soda, two ounces; water, one ounce; olive oil, four ounces; flowers of sulphur, four ounces. Dissolve the subcarbonate in the water, and add the oil, so as to make a soap; then add the sulphur by little and little, carefully mixing it. Of this, two ounces are to be used for each friction, and these to be employed twice a-day. Mean period required for the cure, $13\frac{7}{10}$ days. This method presents the advantage of an oil and alkali united in such proportions as to form a soap, by which means it is prevented from staining the linen, and cures the eruption without irritating the skin. It is not without smell. It is suggested that camphor might be substituted for the sulphur, in the proportion of four drachms to the quantity above mentioned.

"4. Sulphureous baths. To a common bath add four ounces of the sulphuret of potass. Mean time required for the cure, $17\frac{3}{10}$ days. This method is very gentle, effecting the cure without inconvenience, but slowly, and not suiting every patient. The bath may be rendered more active, and the cure more speedy, by adding a little sulphuric acid. It is expensive, however, and can scarcely be employed but on a great scale."

---

* See Journal Generale de Medecine, Juillet, or London Med. and Physical Journal, for November, 1824.

From the foregoing it would appear that the itch is rather more obstinate on the Continent than in this country, where, certainly, the cure, in general, is not so tedious as in the above report. It appears, after all, that "*there is nothing like sulphur.*"

---

7 *Hydrophobia.* Dr. Booth, of Birmingham, (see Bibliographical Record) has recently proposed the injection of acetate of morphine into the veins, as the most probable means of checking this dreadful disease, where the neglect of excision has permitted the virus to affect the system. This *proposal* is founded on the well known case published by Magendie and others—which case is now universally allowed not to have been hydrophobia at all—at least it was not from the bite of a rabid animal, but a spontaneous disease *resembling* rabies canina. So far the proposal rests on a bad foundation. But whoever examines the body of a person who dies of real rabies canina will be convinced that there is little or no chance from any remedy, after the symptoms have once commenced. This, at least, is the impression on our own minds, after opening or assisting to open two unfortunate victims of hydrophobia.

---

8. *On the Utility of Sanguineous Evacuations in the Diseases of Old People.* By J. B. Foucart, M.D. (*Archives for July and September*, 1824.)

It has long been known, in this country, that old people bear bleeding much better than one would, *à priori*, imagine. Sir Anthony Carlisle, we recollect, particularly insists on this point, in his work on old age, and we find that even in France, where the lancet has long been held in such terror, the antiphlogistic treatment begins to gain ground. Our present author was struck with the want of success attendant on the old modes of administering tonics and stimulants in the diseases of old age, and was thereby led to an opposite system. In this system he was more fortunate, and has related a number of cases in illustration. But as these cases are necessarily successful ones, and as the treatment adopted, even by M. Foucart, is less energetic than that employed by the best practitioners in this country, we shall not go into the paper in this place. It is for pathology we look to our Continental neighbours.

---

9 *Hydrocephalus.** A case of this kind, related by Dr. Henry Davies, of Conduit-street, the able physician accoucheur of the Western Dispensary, &c. is calculated to afford hope, even under circumstances apparently desperate.

*Case.* A male child, born in May, 1823, was placed with a wet nurse for six months, after which he appeared to thrive very well till

* Dr. Henry Davies, Med. Repos. March 1825.

November; but his appetite was ravenous. In December he had an attack of diarrhœa and vomiting, which were relieved. In January 1824, he had another bowel complaint, with fever and slight convulsions, extreme irritability and restlessness, short cough and dyspnœa. The inflammatory symptoms were relieved by leeches, mercurial purgations, blisters, evaporating lotions to the head. The child, however, became weak, lost its sensibility, and its head enlarged. The sagittal suture expanded from the root of the nose to the vertex:—the anterior fontanelle was from two to three inches in diameter, and the coronal suture expanded also to some extent." The pulse was excessively quick. *Two grains of calomel and ¼ grain of digitalis every four hours. Two drachms of mercurial ointment to be rubbed in daily. January* 14. Great emaciation—all the symptoms of hydrocephalus. *One grain of calomel, squill, and digitalis night and morning.* A cordial mixture, with ammonia and some tincture of opium, every 4 hours. A little white wine whey to be allowed. 21st. He was purged much, and made large quantities of urine. Relieved a little by the whey. 28th. Better in all respects—tongue and mouth cleaner—makes water very freely—pulse fallen to 112—very little salivation. The powder to be taken only at night—the frictions to be continued. February 2d. Improving still. The powders and mercurial frictions to be continued. The two drachms of strong ointment were rubbed in by the mother for three weeks, (her hand being guarded by a bladder) and it was subsequently used in smaller quantities. The head became reduced almost to its natural size. From this time, with some occasional relapses, he progressively recovered, and one year after the commencement of treatment, was considered as well—his head, however, not being quite reduced to its natural size. "What tended very much," says Dr. D. "to favour the fortunate result of the case, was the ready expansion of the cranium, by which pressure on the substance of the brain was avoided, or very much diminished." It is amazing, as Dr. D. justly observes, to what an extent mercury may be used in diseases of children, without producing its usual effects—salivation. "It evidently, in this instance, exerted its influence on the absorbents, its action being aided by the diuretics combined with it."

"The recuperative power in infants is very great; and this should encourage us not to give them up too soon. There is a time, when disease has lost its hold, and when the patient is in a state of collapse, that stimulants now and then exert a most beneficial influence. The combination of volatile alkali and opium, under such circumstances, seems well adapted as medicine, and white wine whey as food. I have seen infants kept alive for days, by giving them one or two drops of the sp. ammon. arom. in a teaspoonful of the breast milk every hour, and supplying artificial warmth, by heated stone bottles in their cots, till they have at last rallied and perfectly recovered." 205.

This case is creditable to the judgment and perseverance of Dr. Davies.

### *Pharmacology.*

1. *Flowers of Colchicum.* Mr. Frost, the ingenious botanist, and now secretary to the Humane Society, has drawn the attention of the profession to the necessity of examining the state of the root of colchicum, or rather the time of the year at which it is collected, before they employ it in medicine. It is well known, though perhaps little attended to by herbalists in general, that vegetables undergo material changes as to the nature of their juices at different periods of the year, and therefore the discordant accounts which we hear respecting the effects of colchicum may often depend on the period of its collection. To obviate all possibility of deception, Mr. Frost proposes that the flowers should be used, as they will afford the profession "a certain, uniform, and safe medicine," while at the same time they will "not produce those unpleasant effects which occasionally follow the employment of the bulb." The collector cannot err in respect to the time of gathering the flowers. A vinegar, wine, or tincture may be made of the flowers. But surely some mistake must have occurred in the formulæ published by Mr. Frost, who directs *one pound* of the flowers to *one pint* of the menstruum.—*Med. Repos. August.*

---

2. *Tartar Emetic in Pneumonia.* Mr. Wood determined to try the Italian method of treating pneumonia with large doses of antimony, in consequence of reading an account of the practice in our cotemporary of the North. A patient soon presented himself, with symptoms of pulmonic inflammation. He was a stout man, 34 years of age. Mr. W. took away 30 ounces of blood, and ordered a mixture containing ℈iij. antim. tartarisat. ʒiss. tinct. camp. comp. and ℥ij. of water. Half of this was directed to be taken immediately, and the remainder in two hours.

*Second day.* Mr. W. found his patient sitting by the fire side. He had spent a restless night. He had taken the medicine as directed. The first dose (30 grains) caused nausea, but no vomiting. The second dose does not appear to have produced any particular effect—at least none are recorded. The pulse was lowered from 104 to 98. The other usual symptoms of pneumonia continued. He was ordered to bed, and a purgative of calomel and jalap prescribed. In the evening the symptoms were rather increased. A mixture was directed, containing four grains of the tartrite of antimony, one drachm of laudanum, and four ounces of water—a fourth part every three hours.

*Third day.* Perspired much in the night—no nausea—the symptoms ameliorated. The antimonial solution repeated, with the addition of two grains of tartar emetic.

The case is too tedious for detail: we shall, therefore, content ourselves with saying, that the patient was obliged to be thrice bled from the arm, and once by leeches—that a copious perspiration was produced by the large doses of antimony, but no vomiting—that, when the medicine was reduced to comparatively small doses, constant nausea, and at last vomiting was produced—that the disease was much longer under treatment than is usual in acute pneumonia—that the medicine proved powerfully *diuretic*, as well as diaphoretic—and, finally, that the case does not hold out much temptation to imitate the practice, though it proves, as far as one case goes, that large doses of tartar emetic are not productive of so much sickness of stomach as small ones.—*Ed. Journal, July*, 1825.

---

3. *Sulphate of Quinine.* This invaluable medicine is daily extending in reputation, and will, we are convinced, soon become one of the most important in the Pharmacopœia. The great draw-back is its excessive price, and therefore we were rejoiced to find announced in one of the foreign Journals, a mode of preparing the quinine and its sulphate which will effect a great reduction of expence. M. Guerette, chief apothecary to the French forces, has discovered that, from bark, after it has yielded the infusion, decoction, and watery extract, almost as much quinine and sulphate of quinine may be obtained, as from bark that has *not* yielded the above officinal preparations. He was led to this discovery by observing that from the aqueous extracts of cinchona, little or no quinine could be obtained; hence it was evident that this valuable substance was left behind in the refuse. It is, indeed, extraordinary that chemists did not see into this before now, knowing as they did, that it required the strongest alcoholic menstrua to extract the quinine from the bark.

A commission has been ordered to examine M. Guerette's memoir, and repeat his experiments, and their report is confirmatory of his discovery. The formula is not yet published by M. Guerette; but we should suppose it cannot differ materially from that employed in obtaining quinine in the common way from unreduced cinchona.

The effect of the discovery on the price of the medicine will soon, we hope, be manifested both on the Continent and in this country.

---

4. *Expulsion of the Tape Worm.** The tænia is a tenant which often sits the summons of the physician, and even resists the most forcible ejectments which the court of pharmacopœia can issue against this rebellious subject.

Dr. Ruggia, a physician at Naples, was induced to try the effects of a decoction of the root of the pomegranate tree, which answered his fullest expectation, by its quick expulsion of the worm. Dr. R's success soon drew the attention of a host of sufferers from all parts of Naples, where the disease is very common, while, at the same time, it roused the enquiry of other medical practitioners, from whom Dr. R. kept his mode of treatment a profound secret. In order to do this more effectually, he caused the patients to come to his own house, and drink the medicine there. A young man, however, whom the Doctor employed to bring the pomegranate root from the country, betrayed his worthy master, and the secret became divulged a few months ago at Naples.

* Dr. Milne, resident physician at Naples

In consequence of this, the remedy was extensively tried at the great hospital, and with complete success. Dr. Milne has also employed the remedy, which answered his most sanguine expectations. The mode in which he administers it is as follows: two ounces of the fresh root of the pomegranate tree are sliced finely, and slowly simmered down from a pint and a half to a pint, and then strained. Of this, one third part is given early in the morning, and another third every two hours, till the whole is taken. If this does not expel the worm, the same is to be repeated the next day, and so on. Some physicians at Naples use the bark of the root only; but Dr. Milne does not appear to think it superior to the root generally.*

---

5. *Hydrophobia.*† This case of Dr. Brandreth's is remarkable for the length of time which intervened between the infliction of the bite and the explosion of the hydrophobia. The unfortunate youth was bitten by a dog decidedly mad, in November 1823, and did not fall ill till the 13th of October, 1824. On the 15th, hydrophobia was unequivocally established. At 2 o'clock on that day, a solution of acetate of morphia was injected into one of the brachial veins, and seemed to be attended with considerable mitigation of the symptoms. This was repeated two or three times. Two moxas were burnt on the pit of the stomach, and a pint of warm water was also injected into the veins. The mitigation of the hydrophobic phenomena was certainly remarkable; but it was only temporary, for the patient expired in strong convulsions the same evening.

*Dissection.* The vessels of the brain were injected, and the sinuses filled with black blood, which, in the lateral sinuses, was coagulated. There was half an ounce of serous effusion in the ventricles, and a considerable quantity of fluid escaped from the theca vertebralis. The cellular membrane about the throat was distended with air, which did not proceed from the lungs. The small intestines were much distended with air, and exhibited marks of incipient inflammation on their surface. The colon was greatly contracted. The upper and outer surface of the stomach was healthy; but on turning it up, a large perforation was discovered, capable of admitting the closed hand, from which a clear-coloured fluid had escaped into the abdomen. The pericardium was highly vascular—the right auricle and ventricle of the heart were distended with coagula—the left empty. The whole pharynx and trachea were highly inflamed, and the back of the throat was covered with bloody, frothy mucus. The lower part of the œsophagus was deprived of its mucous membrane. There were many spots of extravasated blood between the coats of the stomach.

Since the foregoing case occurred, our author had an opportunity of examining the stomach of a dog that died rabid. It presented appearances precisely similar to those in the human stomach just described.

---

* Dr. Johnson has received some of the root from Dr. Milne, and will supply any gentleman in town with some of it, for trial in tænia.

† Dr. Brandreth. Ed. Journal, January, 1825.

Our author is confident that the acetate of morphia "mitigated the sufferings of the patient in a most decisive manner;" but then again he says—"it is not improbable that the injection contributed to produce the highly vascular state of the intestines, stomach, trachea, and the contents of the head—and that it did so, may be inferred from Magendie having found the same circumstance take place in healthy dogs, into whose circulation opium had been injected." If this be the case, the remedy seems as bad as the disease.

---

6. *Erysipelas—Ol. Terebinth.* Mr. Cox has related (Med. Repos. April) a case of erysipelas where life was apparently in great danger, from cerebral affection, and where the oil of turpentine seemed to have a very beneficial effect. The patient was a female, 21 years of age, who became affected with erysipelas of the scalp, face, and breast, attended at first with restlessness and delirium—afterwards with coma and insensibility. Many other symptoms portending a fatal event were also present. She had been five days insensible, when Dr. Copland recommended the oil of turpentine combined with castor oil, both by the mouth and per anum. The turpentine procured several offensive stools—the pulse lost some of its rapidity—and the coma was rendered less profound. The medicine was repeated for some days, and the patient was gradually restored to convalescence, and ultimately to sound health.

---

7. *Action of Calomel in Indian Diseases.* In the last No. of the Edinburgh Medical and Surgical Journal, there is an extract from "the Minutes of a Meeting of the Medical and Physical Society of Calcutta," which conveys the gratifying intelligence that our brethren of the East are shaking off the mental torpor engendered by the climate in which they live, and that they are likely soon to make a respectable contribution, in the shape of a volume of transactions, to the general current of medical literature and science. When we speak of "mental torpor," we are very far from insinuating that there is any original defect of energy among our Eastern brethren. They are constituted like other people; but they are placed in a situation little calculated to call forth the native powers of mind or body. What, we would ask, are the great whet-stones (if we are permitted such an expression) of human intellect—the great goads to human industry? The collisions of opinion, the clashings of interests, the dread of poverty. What operation can these have among our brethren, scattered as they are over the widely distant stations of the East, where the eternal monotony of Indian life, the exhausting fervour of a tropical sun, and the cheerless gloom of "hope deferred," conspire with the abstraction of all the above-mentioned stimuli, to sink both mind and body into listless languor and indifference! Under such circumstances, it is wonderful that so much has been done on the burning shores of the eastern world, and we are always ready to hail the labours of our oriental cotemporaries as proofs of more than common native talent, energy, and zeal.

Till we have the pleasure of receiving the promised volume, we shall content ourselves with the following extract, because it bears on a subject now under discussion in Europe—namely, the disproportioned effects resulting from large and small doses of medicines. Every one, at first, disbelieved that a scruple or half a drachm of calomel could be taken without hyper-catharsis—and still more that the same quantity of tartarized antimony might be swallowed without hyper-emesis. Yet these facts cannot now be denied, though their correctness seems still to be doubted.

"Mr. Annersley commences his paper, by remarking it as singular, that after so many years' experience in the use of calomel, and the great diversity of opinion yet prevailing on the subject, no investigation should have been entered upon with a view to ascertain the direct effects of this medicine on the stomach and bowels, and the alterations it gives rise to in their secretions. He then proceeds to state his mode of administering the remedy, which was at one time, to give moderate quantities; but on perusing Dr. Johnson's work, he was induced to alter his practice, and to substitute 20 grains for his former doses. The diseases he commonly employs it in are fever, dysentery, and hepatitis; and he gives it in combination with two grains of opium every seven or eight hours, followed up by a brisk purgative. The mouth should never be affected; and when this takes place, Mr. A. conceives the salutary operation of calomel is interrupted. With respect to a prevailing belief, that many constitutions in India are ruined by the use of this drug, if there be any truth in it, Mr. A. is disposed to attribute such deleterious influence to perseverance in small doses, after the necessity for using the medicine has ceased. His opinion is, that a large dose of calomel acts as a sedative. With reference to ascertaining its direct effects, Mr. A. instituted a series of experiments on dogs, administering the drug to them in doses of one, two, and three drachms; and after a definite period, killing the animals, and inspecting carefully the stomach and intestines. The results he considers highly satisfactory; and they lead to the inference, that the natural state of the stomach and intestinal canal is high vascularity; and that the operation of the calomel in large doses is opposed to inflammatory action. Hence is explained in some degree the effect of scruple doses of calomel in allaying irritability of stomach and vomiting, a circumstance which Mr. A. has often witnessed with astonishment. The operation of calomel on the secreted matter of the intestines is both chemical and mechanical. It renders this more fluid, and imparts to it a blackish grey colour. The former property, Mr. A thinks, may explain the benefit derived from calomel in obstructions of the *ductus communis*. He makes some observations also on the change of colour produced by the admixture of calomel with cystic bile; and concludes the paper by expressing a hope that the experiments detailed may lead to a further investigation of the subject, and induce his fellow members to turn their attention towards it."—*Ed. Journal, July, p.* 215.

